# Impact of Short-Term Abstinence on Semen Quality and Oxidative Stress in Men with Suboptimal Semen Parameters: A Prospective Observational Study at a Tertiary IVF Centre

**DOI:** 10.5935/1518-0557.20250159

**Published:** 2026

**Authors:** Garima Sachdeva, Arveen Vohra, Mohammed Ashraf, Devi Ravikumar, Rahul Choudhary

**Affiliations:** 1 Milann Fertility Centre, Bangalore, India; 2 St George’s University Hospitals NHS Foundation Trust, London, UK

**Keywords:** short abstinence, semen analysis, *in vitro* fertilization, DNA fragmentation index, reactive oxygen species

## Abstract

**Objective:**

The study aimed to evaluate the effect of short abstinence on the semen parameters and reactive oxygen species (ROS) levels. A secondary aim was to assess the value of the CANros test for ROS assessment and any correlation between the DFI determined by CANfrag.

**Methods:**

Thirty patients undergoing infertility treatment provided two semen samples: one after a normal abstinence period (2-7 days; control) and one after a short abstinence period (4 hours; case). Semen analysis included volume, total sperm count, motility, and liquefaction time while ROS levels were measured by the CANros test. Spearman’s rank correlation was made between the results of CANros vs DFI measured by CANfrag and a cost-benefit analysis between the two tests.

**Results:**

There was no significant difference in the liquefaction time between the two samples. However, samples after 2-7 days of abstinence showed higher volume and total sperm count, though the 4-hour samples exhibited better motility. The CANros test was normal in 33.33% of 4-hour samples compared to 16.66% in the 2-7 days samples, though it was not statistically significant. A positive correlation was observed between CANros and DFI results (r=1.0, p=0.0), with the CANros test being more cost-effective.

**Conclusions:**

Short abstinence enhances semen quality by improving motility and reducing oxidative stress. The CANros test is a reliable and cost-effective alternative to DFI testing for evaluating oxidative stress in semen.

## INTRODUCTION

Most of the andrology laboratories have universally adopted the World Health Organization (WHO) protocol of abstinence (2-7 days) when collecting semen samples (Boitrelle *et al.*, 2021). WHO recommends 2-7 days of abstinence to collect semen samples (WHO, 2021). Long abstinence leads to the build-up of spermatozoa in the epididymis, and it may increase their exposure to the harmful effects of reactive oxygen and nitrogen species (ROS and RNS) generated mainly by granulocytes during maturation and storage in the epididymis (Agarwal *et al.*, 2016a). Thus, spermatozoa are susceptible to oxidative attack, which has been correlated with decreased sperm motility, lipid peroxidation, DNA damage, and compromised fertilization rates (Aitken *et al.*, 2012). Standard semen analysis does not identify sperm senescence or functional impairment (Ko *et al.*, 2014). Examining the effect of abstinence at the functional level requires more sensitive sperm tests such as DNA fragmentation and ROS can be detected by the CANros test (microptic, 2025). So, we postulate that a shorter period of abstinence (4hrs) will shorten the transit time of the spermatozoa through the epididymis. This will likely decrease ROS in patients with suboptimal semen parameters, resulting in better pregnancy outcomes.

## MATERIAL AND METHODS

This prospective observational case-control study was conducted at a tertiary IVF unit (Milann - The Fertility Centre) over one year (June 2020 to June 2021). The study aimed to compare the effects of short abstinence (4 hours) versus normal abstinence (2-7 days) on semen parameters and reactive oxygen species (ROS) levels in patients with suboptimal semen quality.

### Inclusion Criteria

- Men under 45 years of age with oligo-astheno-teratospermia requiring intracytoplasmic sperm injection (ICSI), based on the latest World Health Organization (WHO) semen analysis parameters (WHO, 2021) (Volume < 1.4 ml, Total Motility < 42%, Progressive Motility < 30%, Normal Morphology < 4%).
- Men with a single deranged parameter, such as oligospermia, asthenospermia, or teratospermia, planned for ICSI.
- Men with varicocele and high DNA fragmentation index (DFI > 25%).

### Exclusion Criteria

- Men over 45 years of age.
- History of any sexually transmitted infections (STIs) or communicable diseases.

Thirty patients with suboptimal semen parameters undergoing infertility treatment were recruited. After obtaining written informed consent, each participant provided two semen samples for freezin quality of the semen varies with the length of the abstinence period. g before ICSI:

1. Group A (Controls): The first sample was collected after normal abstinence (2-7 days).
2. Group B (Cases): The second sample was collected after a short abstinence (4 hours).

Changes in semen parameters and ROS levels were assessed in both samples.

### Semen Collection Procedure

Participants were instructed to:

- Pass urine.
- Wash hands and penis with soap to reduce contamination risk.
- Rinse away soap.
- Dry hands and penis with a fresh disposable towel.
- Ejaculate into a sterile container via masturbation.

Emphasis was made on collecting the complete semen sample, with any loss of fraction was reported. Details such as the participant’s name, identification number, period of abstinence, completeness of the sample, any difficulties during collection, and the interval between collection and analysis were recorded. The specimen container was maintained at ambient temperature (20-37°C) to prevent temperature-induced spermatozoa damage and was placed on an inc

### ROS Evaluation

The level of reactive oxygen species in the seminal fluid was measured by using CANros kits. This test is based on the reduction of nitroblue tetrazolium dye to determine the total ROS produced by leukocytes and spermatozoa. When this reagent reacts with free radicals in the seminal sample, it produces a color that varies from light pink (normal ROS) to dark purple(severe ROS), according to the concentration of free radicals present in the sample (microptic, 2025).

### Procedure

- The sample is collected in a sterile semen collection jar and is allowed to liquefy at 37°C.
- If the sample is too viscous, liquefaction of the sample is done using a dropper without creating any bubbles.
- The test shall be done within 30 minutes of sample collection.
- The Agarose-N-Gel tube in the kit is boiled in 90-100°C water for 2 minutes or till the gel melts. Immediately after melting, the gel tube is kept for 5 minutes at 37°C.
- 200 µL of semen sample is then added to this melted Agarose-N-Gel tube and mixed smoothly without bubble formation with minimal stress on sperm cells.
- Incubation of the tube is done at 37°C for 55 minutes.
- The color change is seen immediately after incubation and is compared with a color code that is provided in the kit to interpret the oxidative stress level of the sample.

The CANros test results are interpreted based on color changes that indicate varying levels of reactive oxygen species (ROS). A white or light pink color signifies a normal ROS level, while a light purple color suggests a low level (mild) of ROS. A purple color represents a moderate level of ROS, and a dark purple color indicates a high level of ROS. This color-coded system allows for easy assessment of oxidative stress levels (microptic, 2025).

The principal outcome measure was the CANros outcome of the two groups. Secondary outcome measures were the comparison of differences in conventional semen parameters such as semen volume, concentration, total sperm motility, progressive motility, normal forms, and the eventual need for invasive sperm retrieval for ICSI.

### Assessment of DNA fragmentation index (CANfrag)

The CANfrag DNA Fragmentation Test Kit was utilized to assess DNA fragmentation in semen samples collected after normal abstinence (2-7 days). This assay, a type of sperm chromatin dispersion test, relies on DNA denaturation to distinguish between fragmented and intact DNA in spermatozoa. Initially, untreated sperm are embedded between a base agarose layer and an inert top microgel. The samples are then treated with an acid, which induces the formation of single-stranded DNA motifs at the sites of breaks. Sequential lysis treatments subsequently remove nuclear proteins. Staining renders the halo and core structures visible under a bright-field microscope. Sperm with minimal or no DNA fragmentation display large halos of intact DNA loops surrounding the central core, while those with fragmented DNA exhibit very small or no halos. DNA fragmentation in sperm can contribute to male infertility-a condition that might not be detected through routine semen analyses such as sperm concentration, motility, and morphology assessments.

The SDF value is calculated using the following equation:

SDF Value (%) = (Number of fragmented sperm observed / Total number of sperm observed) × 100

### Statistical analysis

Semen sample characteristics from 30 samples were analyzed. For statistical analysis, PASW (SPSS) 22.0 for iOS was used. First, the Kolmogorov-Smirnov test was applied to check the normality of data distribution. For normally distributed variables, a paired Student’s t-test was used, while for the not normally distributed, the Wilcoxon signed-rank test was applied. Spearman rank correlation was used to establish the correlation between the CANros and CANfrag tests. The level of significance taken was α=0.05.

## RESULTS

Liquefaction time showed no significant difference between the samples (4 hours *vs*. 2-7 days of abstinence). Semen samples collected after 2-7 days of abstinence showed an increased volume and total sperm number compared with samples collected after 4 hours (*p*<0.001; Table 1).

**Table 1:** Semen analysis results comparing samples collected after abstinence periods of 4 hours and 2-7 days. Liquefaction time, semen volume, and motility parameters were analyzed using a paired Student’s t-test, with results expressed as mean ± standard deviation (95% confidence interval). Sperm concentration, total sperm count, normal sperm morphology, and round cell concentration were assessed using the Wilcoxon signed-ranks test and are presented as medians with interquartile ranges (IQR: first quartile [Q1] to third quartile [Q3]).

	Group B (4 hours) (n=30)	Group A (2-7 days) (n=30)	p value
**Liquefaction time (min)**	29.1±12.5 (25.6; 32.5)	29.5±12.2 (26.1; 32.9)	0.866
**Volume (mL)**	2.8±1.2 (2.5; 3.2)	3.7±1.6 (3.2; 4.1)	<0.001^*^
**Sperm total motility**	60.5±12.3 (57.1; 64.0)	57.5±11.9 (54.2; 60.8)	0.026^*^
**Progressive motility (%)**	55.1±12.3 (51.7; 58.5)	52.0±12.1 (48.6; 55.3)	0.025^*^
**Non-progressive motility (%)**	5.4±2.8 (4.6; 6.2)	5.5±2.2 (4.9; 6.1)	0.781
**Immotile (%)**	39.5±12.3 (36.0; 42.9)	42.3±12.1 (38.9; 45.6)	0.041^*^
**Sperm concentration (×10^6^/mL)**	53.4; 57.3 (20.4-77.7)	45.2; 76.5 (19.6-96.1)	0.337
**Sperm total number (×10^6^/ejaculate)**	120.1; 168.0 (39.7-207.7)	147.5; 240.2 (74.6 -314.8)	<0.001^*^
**Sperm normal morphology (%)**	6.0; 2.0 (5.0-7.0)	5.0; 3.0 (4.0-7.0)	0.395
**Round cell concentration (×10^6^/mL)**	0.8; 1.0 (0.5 -1.4)	0.7; 1.0 (0.5 -1.5)	0.273

Conversely, samples collected after 4 hours of abstinence exhibited improved motility compared with those collected after 2-7 days (sperm total motility, *p*=0.026; progressive motility, *p*=0.025; immotility, *p*=0.041; Table 1).

### Comparison of CANros Parameters

The CANros test demonstrated a 50% variation in normal results, with 33.33% of samples showing normal values after 4 hours of abstinence, compared to 16.66% after 2-7 days of abstinence. However, this difference was not statistically significant (Tables 2 and 3).

**Table 2:** Comparison of CANros Results Between Short (4 hrs) and Normal Abstinence (2-7 days).

CANros	Group B (4 hours) (n=30)	Group A (2-7 days) (n=30)
**NORMAL**	10	5
**MILD**	8	8
**MODERATE**	7	8
**SEVERE**	5	9

**Table 3:** Comparison of CANros Results Between Short (4 hrs) and Normal Abstinence (2-7 days).

CANros	Group B (4 hours) (n=30)	Group A (2-7 days) (n=30)	p-value (chi-square test)
**NORMAL**	10 (33.33%)	5 (16.66%)	0.095547
**SEVERE**	5 (16.66%)	9 (30%)	

### Correlation between CANros and CANfrag (Oxidative stress and DFI)

**Figure 1:**
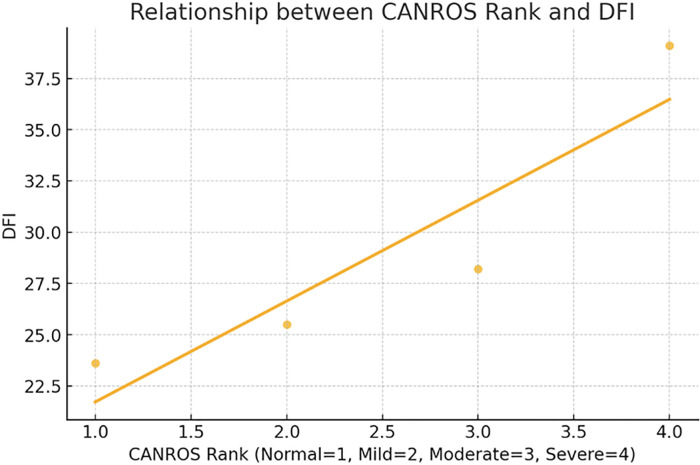
The scatter plot with a regression line shows a clear positive trend between CANros rank and DFI. This confirms the strong relationship between these variables.

The Spearman’s correlation coefficient between CANros (oxidative stress) and CANfrag (DFI) is 1.0, with a *p*-value of 0.0. This indicates a perfect positive correlation, meaning that as the severity level of CANros increases, DFI also consistently increases (Figure 1 and Table 4).

**Table 4:** Spearman’s Rank Correlation Calculation Table. The correlation coefficient of 1.0 indicates a perfect positive monotonic relationship between CANros severity and DFI.

CANROS	DFI	CANROS Rank	DFI Rank	Difference *(d_i_)*	Difference Squared *(d_i_)^2^*
**Normal**	**23.6**	**1**	**1**	**0**	**0**
**Mild**	**25.5**	**2**	**2**	**0**	**0**
**Moderate**	**28.2**	**3**	**3**	**0**	**0**
**Severe**	**39.1**	**4**	**4**	**0**	**0**

### Cost comparison between CANros and CANfrag kits

Testing for oxidative stress using CANros is cheaper than testing for DNA fragmentation index using CANfrag (Table 5).

**Table 5:** Cost comparison between CANros and CANfrag kits.

Kits	Cost	Tests/kit	Cost per test
CANros	Rs- 3920/-	10	Rs- 392/-
CANfrag	Rs- 21000/-	10	Rs- 2100/-

## DISCUSSION

The seminal parameters may vary from person to person and for samples taken over different periods (Baker *et al.*, 1981). To minimize these variations, cases themselves served as their controls, and two samples were taken from the same person, the first one with a 2-7 days gap and the other one obtained with 4 hrs of abstinence on the same consultation day.

Sperm quality and oxidative stress are closely related as several studies have focused on these aspects (de Lamirande *et al.*, 1997). This research work, therefore, sought to assess semen qualitative characteristics along with CANros parameters concerning oxidative stress for the abstinence duration of 4h and 2-7 days.

Conventional semen analysis revealed that samples collected after 2-7 days of abstinence demonstrated higher semen volume and total sperm count compared with those collected after 4 hours of abstinence. These findings are consistent with trends in earlier studies reporting increased sperm accumulation over longer periods of abstinence (De Jonge *et al.*, 2004; Levitas *et al.*, 2005; Lehavi *et al.*, 2014; Agarwal *et al.*, 2016a; Mayorga-Torres *et al.*, 2016; Alipour *et al.*, 2017; Hanson *et al.*, 2018). Conversely, there was significantly higher motility when samples were obtained after 4 hrs. of abstinence, suggesting the possibility that storage times are shorter might better preserve motility (Mayorga-Torres *et al.*, 2016; Alipour *et al.*, 2017).

In several previous investigations, oxidative stress is seen to link up with defective motility (Iwasaki & Gagnon, 1992). Therefore, on identical lines, an increase in motility in longer abstinence samples may be lower due to a rise in the level of oxidative stress in this study.

ROS, at physiological levels, plays a part in sperm functions such as maturation, capacitation, hyperactivation, acrosome reactions, and successful fertilization (Aitken *et al.*, 2012). On the other hand, an imbalance in generation and antioxidant defenses is a source of oxidative stress that causes oxidative damage to spermatozoa (Venkatesh *et al.*, 2011; Aitken *et al.*, 2012). Long storage of sperm within the epididymal tail exposes them to ROS at an increased potential for lipid peroxidation, mitochondrial dysfunction, DNA damage, and acrosomal impairment (Hanson *et al.*, 2018; Boitrelle *et al.*, 2021).

Results from the CANros test showed higher oxidative activity in samples after 2-7 days of abstinence, which reflects prolonged exposure to ROS. Under these conditions, ROS production is contributed mostly by spermatozoa themselves, as leukocytopenia was excluded in this study. High intracellular levels of ROS may reduce motility and impair functional integrity, as reflected by the significantly lower motility in these samples (Said *et al.*, 2004).

There are various tests described in the literature to test oxidative stress in semen like the nitro-blue tetrazolium dye test (Esfandiari *et al.*, 2003; Agarwal *et al.*, 2016a) (calorimetric test like CANros), chemiluminescence assay, cytochrome c reduction test, fluorescein isothiocyanate (FITC)-labeled lectins, and electron spin resonance (ESR). The Nitroblue Tetrazolium (NBT) test (CANros) is recognized for its cost-effectiveness and user-friendly approach to detecting free radicals, especially reactive oxygen species (ROS) generated by neutrophils (Agarwal *et al.*, 2018). While chemiluminescence assays offer high sensitivity and specificity, they often necessitate sophisticated equipment and larger sample volumes, making them less accessible for routine applications (Agarwal *et al.*, 2016a). In contrast, the NBT test (CANros) provides a straightforward alternative by visually indicating ROS levels through observable color changes. Unlike the cytochrome c reduction test (Dikalov & Harrison, 2014), which is adept at quantifying superoxide anions (O₂•⁻) but may face challenges with low enzymatic activity and is not suitable for detecting intracellular ROS, the NBT test (CANros) offers a practical solution. Additionally, methods employing fluorescein isothiocyanate (FITC)-labeled lectins (Aitken *et al.*, 2012; Farah *et al.*, 2013; Boitrelle *et al.*, 2021) are effective for assessing acrosome status but can encounter difficulties in distinguishing between true and false acrosomal reactions. Furthermore, electron spin resonance (ESR) is versatile for quantitative and kinetic analyses of free radicals but may be constrained by interference factors and the need for specific spin-trapping agents (Kohno, 2010; Dikalov & Harrison, 2014). Overall, the NBT test (CANros) stands out as a preferred choice when simplicity and cost-efficiency are essential, though it’s important to note that subjective interpretation can influence result accuracy.

The assessment of oxidative stress in seminal plasma can be performed by using indirect tests like the Endtz/myeloperoxidase test, measurement of lipid peroxidation, chemokines, antioxidants/ micronutrients/vitamins, ascorbate, total antioxidant capacity or DNA damage using indirect laboratory methods (Esfandiari *et al.*, 2003; Reuter *et al.*, 2010; Sharma *et al.*, 2010; Agarwal *et al.*, 2016b; 2018; Roychoudhury *et al.*, 2016; Bisht *et al.*, 2017). In this study, we employed the CANfrag test, which assesses DNA damage based on sperm chromatin dispersion (Fernández *et al.*, 2003). DNA assays are more complex and susceptible to variability, whereas the NBT (CANros) test provides a quicker and simpler method, though it lacks specificity for DNA damage (Agarwal *et al.*, 2018). This study indicates a positive correlation between oxidative stress testing (CANros) and DNA damage testing (CANfrag). However, CANros serves as a more cost-effective alternative to CANfrag.

## CONCLUSION

Short abstinence enhances semen quality by improving motility and reducing oxidative stress. The CANros test is a reliable and cost-effective alternative to DFI testing for evaluating oxidative stress in semen. These results may be considered to obtain better results in assisted reproduction techniques to improve both fertilization and implantation rates.
